# Cybersecurity as it relates to perfusion

**DOI:** 10.1051/ject/2025064

**Published:** 2026-03-13

**Authors:** Kara Lung

**Affiliations:** 1 Boston Children’s Hospital Boston MA USA

**Keywords:** Perfusion, Cybersecurity, Health Policy, Data Security, Education, Healthcare Infrastructure

## Abstract

Perfusionists must maintain strong digital security habits, know the inherent risks of devices in use, and have a healthy respect for the consequences of a security breach at a hospital. While perfusion has largely been able to operate without much interaction with cybersecurity experts, the relentless advancement of the digital age means that perfusionists cannot remain oblivious to the intersection of their devices and hospital digital security. This article provides a historical overview of healthcare cybersecurity with specific recommendations for perfusion teams looking to ensure best practices for protected health information (PHI). Critical recommendations include keeping physical copies of downtime procedures, routine practice of downtime procedures, discussion with the hospital information technology (IT) team to confirm perfusion-based asset lists, and the creation of an American Society of Extracorporeal Technology (AmSECT) standard or guideline regarding attention to cybersecurity.

**Abbreviation**
AHA: American Hospital Association
CISA: Cybersecurity & Infrastructure Security Agency
DDoS: Distributed Denial of Service
DHHS: Department of Health and Human Services
ECMO: Extracorporeal Membrane Oxygenation
EMR: Electronic Medical Record
FDA: Food and Drug Administration
FDORA: Food and Drug Omnibus Reform Act
HIPAA: Health Insurance Portability and Accountability Act
HITECH: Health Information Technology for Economic and Clinical Health
HLM: Heart-Lung Machine
IoMT: Internet of Medical Things
IoT: Internet of Things
IT: Information Technology
KEV: Known Exploitable Vulnerability
MDM: Medical Device Manufacturer
NIST: National Institute of Standards and Technology
SBoM: Software Bill of Materials

## Introduction

Cybersecurity is the safeguarding of computer systems, networks, and electronically stored data from both disruption and unauthorized access, use, or disclosure [1, 2]. Cybersecurity for healthcare systems has long been a complex challenge for a variety of reasons, including unsecured medical devices, patchwork networks, complex governmental oversight, limited institutional funding, and breadth of hospital networks. Electronic medical records (EMRs) sit at the heart of modern healthcare systems, consolidating data from a myriad of human and electronic sources. EMRs support the decision-making efforts of clinicians, smooth workflows, and increase communication between individuals, departments, and hospitals [3]. The tradeoff with increased interconnectivity of devices is a larger risk of data breach, which in turn jeopardizes patient privacy, patient financial stability, patient quality of care, and hospital operations [4].

Fundamental to the understanding of the recent technological advancement of healthcare is the understanding of the Internet of Things (IoT). The IoT is a collection of physical devices that all have the ability to collect and process data, and exchange this data when connected to a network [5]. Together, they form a web-like structure of information transfer. Through this structure, one vulnerability in any single device’s security can affect many other devices and services [6]. When the IoT is composed of medical devices, it is referred to as the Internet of Medical Things (IoMT), and it is responsible for a lot of improved monitoring, care, and communication, particularly in data-rich environments such as the intensive care unit [7]. Infusion pumps, pacemakers, ventilators, electrocardiography machines, pulse oxygen monitors, and hemodynamic monitors are all examples of devices connected to the IoMT. For perfusion, this list can include heart-lung machines (HLMs), in-line blood gas monitors, cell salvage systems, point-of-care analyzers, near-infrared spectroscopy, bispectral index monitors, extracorporeal membrane oxygenation (ECMO) machines, balloon pumps, and more, some of which are essential to survival. Each connected device brings an additional access point onto the hospital network, increasing the metaphorical surface area on which a cyberattack can occur [5]. As such, the IoMT is currently one of the largest threats to hospital security [7].

Since 2014, there has been an explosion of research related to the field of cybersecurity, and with it, a discussion of why healthcare is unique in its vulnerabilities. Healthcare is one of the most targeted sectors for cybercrime due to being both high in value and relatively low in defense [8]. Healthcare records have been noted time and again as the most valuable type of personal information on the dark web. A complete set of health data can be sold for ten to a hundred times more than stolen credit card information [3, 5, 6, 9–11]. Unfortunately, once health data is compromised, it is not possible to restore a victim’s privacy or undo the psychological harm done [3]. Considering the high value of healthcare data, cybersecurity is a necessity for all hospitals and perfusion teams.

For hospitals, there are a multitude of challenges associated with implementing cybersecurity. There are legacy devices, systems that are critical and must remain in use, and substantial time and resources necessary to implement cybersecurity, which can be disruptive to workflow [1]. Oftentimes, healthcare cybersecurity is seen as a compromise between patient safety and information security, and as one might expect in a patient-focused system, patient care is often prioritized at the expense of digital security [12].

Healthcare workers also tend to have a low understanding of cybersecurity and the risks that their organization faces from cyberattacks [13]. Educating employees on the importance of cybersecurity and empowering them to maintain organizational security can be one of the most successful strategies for improving cybersecurity. This is particularly effective in small organizations that may lack a well-staffed IT security team [2]. Properly emphasized, cybersecurity education contributes to the security culture of an organization, which is critical against the ever-evolving tactics of cyber criminals [2]. This article provides a historical overview of healthcare cybersecurity with recommendations for perfusion teams looking to ensure best practices for protected health information.

## Background

Healthcare cybersecurity is a mix of governmental oversight, software applications, medical devices, third-party vendor connections, and human actions [2]. The term “cybersecurity” can include more than just defense against cyberattacks. It also covers accidental or internal breaches of data that are not the result of an external force. In fact, 70% of all data breaches are the result of employee carelessness [12], and up to 95% of breaches within the healthcare field are from human error [2, 10, 13]. The cost of a healthcare data breach is the highest of any industry, and healthcare has held that title since 2011. In 2023, the average healthcare data breach cost $9.77 million, while the next costliest industry, finance, cost $6.08 million per breach [14]. In 2016, it was estimated that 90% of hospitals had had one or more data breaches, and 45% had had five or more [1]. The Poneman Institute also estimated that in 2016, there was $6.2 billion lost per year due to healthcare data breaches [12]. Some of this is due to the penalties imposed upon hospitals by the US Department of Health and Human Services’ (DHHS) Office of Civil Rights for data breaches affecting over 500 individuals, but a much greater portion of that comes from the cost of investigation, remediation, and identity protection of affected individuals after a breach has occurred [6, 15].

Cyberattacks in general have been increasing in quantity over the past decade [5], and in 2019, 24% of all cyberattacks were against healthcare [1]. The US DHHS reports that across all industry sectors, there have been an average of 4,000 ransomware attacks per day since 2016 [16]. Digitization of patient health records has only increased the scale of impact a data breach can have on a hospital and its patients [8].

### Historical attacks on hospitals

The rapid increase of reported cyberattacks on hospitals has been well documented [6]. In 2015, the healthcare giant Anthem experienced an attack that exposed 78.8 million patients’ data, a record for the 2009–2019 timeframe [7]. In 2016, Hollywood Presbyterian Hospital was shut down for 10 days after being hit by a ransomware attack that likely began from a phishing scam [6]. They eventually paid $17,000 in bitcoin to the attacker in the first highly publicized ransomware attack on a hospital [6, 11].

2017 was notable for the WannaCry attack that took out 50 National Health Service (NHS) hospitals in the United Kingdom, even though the cyberattack was not initially directed at the healthcare system [6, 17]. This attack is famous for using a weakness of the unsupported Microsoft XP operating system, which had been discontinued in 2009. WannaCry resulted in almost 20,000 canceled appointments and cost £92 million (118 million USD) in lost revenue over four days [7]. By 2020, it was reported that nearly 94% of global healthcare institutions had experienced some variety of cyberattack or data breach [9].

In 2021, a ransomware attack on the National Healthcare Network of Ireland took out all clinical and non-clinical systems [13]. A third-party website tracking device on a patient portal website caused a data breach for Advocate Aurora Health in 2022 [18]. Community Health Systems was hit with ransomware in 2023, resulting in around one million patients’ data being stolen [18]. Lurie Children’s Hospital was hit with ransomware in 2024 [19], as was Boston Children’s Health Physicians group later that year [20].

It is not just hospitals and associated systems that get attacked, but also third-party vendors like LivaNova, which were attacked in November of 2023, compromising patient data [21]. The nonprofit blood bank OneBlood was hit with ransomware in 2024 [22]. The Red Cross was hacked in 2022 [23], and UnitedHealth Group’s subsidiary Change Healthcare, which primarily worked with payroll and hospital finances, was hit in early 2024. Change Healthcare’s ransomware attack may impact up to a third of all Americans, and as of September 2024, it has cost at least $2.3 billion in remediation and repair [24]. See Table 1 for more examples.

**Table 1:** Common types of malware, their effects, and examples.

Category	Effect	Recent Example Healthcare Victims
Ransomware	Denial of a system or database, generally through encryption, until a ransom is paid for access restoration.	Ascension (2024) [18]
		Change Healthcare (2024) [18]
		National Healthcare Network of Ireland (2021) [13]
		Regal Medical Group (2023) [18]
		Community Health Systems (2023) [18]
		WannaCry (2017)
		Hollywood Presbyterian Medical Center (2016) [6, 11]
		Lurie Children’s Hospital (2024) [19]
		Change Healthcare 2024 [24]
Destructive extortion	Similar to a ransomware attack, save that once the target system is encrypted, data is deleted or the system is destroyed on purpose.	Hancock Regional Hospital (2018) [3]
Denial of Service (DoS) or Distributed Denial of Service (DDoS) [25]	Digital services are overwhelmed by excessive network traffic, thereby limiting the amount of legitimate electronic requests that get through to the intended recipient. Attack can originate from a single or multiple (distributed) sources.	Boston Children’s Hospital (2014) [26]
Man-in-the-Middle (MITM) [9]	Information passes through an additional process that reads or copies the information to a third party. Data integrity can easily be compromised in this situation.	St Jude Merlin@Home pacemakers [27]
Abuse of known software vulnerabilities	Use of known and unpatched vulnerabilities in software to gain access to a system.	Epiphany Cardio Server SQL injection (2015) [28]
		Medical Informatics Engineering SQL injection (2015) [18]
		South-Eastern Norway Regional Health Authority (2017) (Legacy Windows XP) [3]
		Red Cross (2022) [23]
Privilege escalation/abuse of privilege [9]	Attacks that have a goal of gaining higher levels of access or privilege so that malware has a broader impact when deployed. It can spread “horizontally” through same-level access points, or “vertically” to more privileged accounts.	Hancock Regional Hospital (2018) [3]
Third-party breach	When a third-party service fails to properly maintain data security. Often, from addition of Google/Facebook or other advertiser tracking algorithms are applied.	Advocate Aurora Health (2022) [18]
		Boston Children’s Health Physicians (2024) [20]

### Historical medical device vulnerabilities

Individual healthcare devices have been reported as vulnerable to cyberattacks as well. These vulnerabilities could serve as gateways to larger networks, in addition to being devastating in their own right. One of the challenges innate to medical devices is the lack of basic securities such as encryption, which can drastically lower the battery life of many devices like pacemakers and insulin pumps. Pacemaker security has been the topic of discussion many times in the media, most notably when former Vice President Dick Cheney had his pacemaker modified after an issue was discovered that would have allowed a cybercriminal to modify the settings or deliver a fatal command remotely [27].

Abbot had a major recall on their St. Jude pacemakers in 2017 after realizing that it could be accessed and modified. In 2023, Medtronic had a major vulnerability reported in their PaceArt Optima system – now a part of PaceMate – where patient data could be exploited as it is compiled and managed. The system was also vulnerable to Denial-of-Service attacks that would slow the data transmission or render the connection unresponsive [29].

Medtronic also had a cybersecurity risk in 2022 for their insulin pumps MiniMed 670G, where they could be wirelessly taken over when pairing with other system components [30]. Infusion pumps such as Hospira Lifecare Drug infusion pumps and GE’s Alaris Gateway Workstations were found to have weaknesses that would allow a cybercriminal to remotely make changes in medication dosing [7, 27]. The Aestiva and Aespire ventilators and anesthetic machines, also by GE, were similarly easily changeable when connected to an unsecured network [7].

Another major issue is devices with hardcoded passwords from the manufacturer. These passwords can sometimes be found in device manuals online, and when devices with vulnerabilities like these are accessible on a network, they are a liability. One example of this type of situation is blood refrigeration units, which could have the alarms disengaged and the temperature modified remotely in order to destroy products [27]. Magnetic resonance imaging equipment and computed tomography (CT) scanners have fallen under similar scrutiny, with concern that cybercriminals could remove the radiation exposure limiter from a CT even while a patient was being scanned [27]. Heating, ventilation, air conditioning, and electrical systems can be targets as well, impacting the physical environment in which staff work and patients are treated [3].

### Healthcare specific vulnerabilities

Healthcare as a whole has a variety of weaknesses that make it difficult to defend. Physically, hospitals are built to help people. Anyone can walk in off the street and expect to be attended to in some way. If someone were to plan malicious cyber activities, it would not be difficult to situate themselves near equipment that has access to the larger hospital network while waiting for medical assistance [4].

From a cyber-perspective, hospitals have difficulty because networks in most hospitals were not built from the ground up with security in mind. Unless a hospital has invested significant funds in revamping its whole system, the network has been built in patches, branches, and add-ons over decades, making it difficult to understand and comprehensively cover. As an example, most hospitals have EMRs, but many also have separate databases and record-keeping services for individual departments or devices. Some of these services would have been slowly absorbed into a larger EMR, while others persist, often still linked to external companies. As an example, heart-lung machines may be linked to external perfusion record companies that may have vulnerabilities in their security and impact perfusionists regardless of what the hospital’s internal IT team is prepared for. For perfusion as a whole, the aspect of IoMT cybersecurity that is of most concern comes from the many devices that we operate and the lack of investment in digital education regarding cybersecurity risks.

### Financial barriers to hospital cybersecurity

Another major factor is the lack of funding that cybersecurity receives within a hospital or hospital system [1, 4, 7]. Hospitals generally run on a 1–3% profit margin, which is significantly lower than the margins available to other businesses [8, 9]. Some of this low profitability is due to fixed insurance reimbursements, which cannot be easily changed to include a fee for future improvements to hospital information networks [4]. Add to this a shortage of IT security specialists [1, 4, 8, 10, 14, 17] and the fact that those specialists can usually make significantly more money working in the private sector, the result is a chronically understaffed and underfunded department doing its best to defend a piecemeal network against threats that the hospital itself may physically allow through its doors.

The breadth of the network, variety of devices connected, and age of individual devices are other issues hospitals face [31]. Medical devices need to be able to transfer data to and through many systems in the hospital, but often do not have proper security measures. Older and smaller devices often do not have the battery life or processing capacity to run encryption, digital forensics, malware detection, or threat modeling processes [3]. Some medical devices run off older and unsupported operating systems like Windows XP, which have known security flaws that can be exploited by cybercriminals [27].

These kinds of older, unsupported, and unpatched devices are often known as legacy devices. They are not removed from hospital systems because they perform critical functions, are too expensive to replace, or there is staff resistance due to workflow concerns or familiarity with the device [1, 4]. Frequently, for hospitals that use devices with old operating systems, such as Windows XP, the cause is that there is either no update available for an individual device, or a particular program can only run on older operating systems. Another reason is the financial burden, as the cost of upgrading systems may not be built into a hospital’s budget. However, a serious risk to a hospital system comes with keeping legacy devices on the network [17]. It is easier for malware to avoid detection on a legacy device, and some cybercriminals will intentionally run old malware to target these systems [3]. Legacy systems are known to contribute to higher incidences of cybersecurity attacks in healthcare [4, 6]. Unfortunately, securing existing devices on a hospital network or replacing old devices with newer and more secure ones is a slow and expensive process, during which patients and hospitals will remain exposed to attacks [32].

The current trend of consolidating hospitals into larger networks also brings cybersecurity risks. Pooling resources may reduce overhead costs, but it also increases the vulnerabilities of many systems through increased access and the larger target that a hospital network presents. While a network of hospitals may increase the funding of an overarching IT team, streamlining software systems, vendors, and services over time, resulting in a reduction of risk, in the short term, a lot of time and money are necessary to overhaul the new network additions, during which there is vulnerability in spades.

### Human barriers to hospital cybersecurity

The most pressing vulnerability inherent to healthcare networks is the human one. The sheer number of end users in healthcare settings, be they patients, visitors, or staff members, complicates network access control [4]. End users are often the weakest link in cybersecurity [10], and in a study in 2021, only 16% of healthcare workers had confidence in their understanding of phishing, a common method that cybercriminals use to gain access to a system [10].

The Poneman Institute showed that employee training, specifically on the recognition of phishing attempts, was the biggest factor that reduced the cost of a data breach, while conversely, the things that increased the cost the most were the complexity of the system, a shortage of IT security staff, and third-party breaches [14]. End users, including perfusionists, are a vulnerable part of the cybersecurity system. Staff burnout, distraction, fatigue, and an excessive number of emails in an inbox can negatively impact a person’s ability to recognize phishing attempts [10, 33]. All of these are common features amongst health professionals. Things that increase a staff member’s ability to handle phishing attempts are time, education, personal experience, and repetition of testing [8, 10]. So while nurses, doctors, and perfusionists are in charge of patient care, cybersecurity is increasingly becoming a patient safety concern that these individuals are not equipped to deal with.

## Why should perfusionists be concerned about cybersecurity?

The ramifications of a cyberattack on a hospital are varied. Depending on the type of attack (Tab. 1), different hospital systems may be affected. In the case of ransomware, a hospital’s data and daily functions are held hostage through encryption of the hospital’s data until a sum is paid to the attackers, usually in a cryptocurrency such as bitcoin. Ransomware is an increasingly common goal of cybercriminals and causes a denial of access to patient information and electronic charting. This may include the perfusion record and intraoperative documentation. Forced downtime like this compromises patient care through lack of access to critical information such as patient allergies, medications, and comorbidities [3]. Perfusionists and other operating room staff who do not regularly use paper charting may have a difficult time adapting on the fly, and the reconciliation of paper into electronic records after the attack is over can also be a significant burden. Incoming patients may be diverted away from impacted hospitals, causing delays in care, and canceled appointments can be difficult to reschedule [2].

One study by Choi et al found that there was an increase in the 30-day mortality rates of acute myocardial infarction (AMI) patients after hospital PHI is breached. Data breaches have been shown to effectively erase a year’s worth of improvements on AMI mortality rates at impacted hospitals [15]. The implementation of remediation can take two to four years to complete, meaning that the time until a hospital’s quality of cybersecurity improves can take that long as well. Meanwhile, the remediation process can involve training time with new systems, which slows the hospital’s workflow in the short term [15]. The faster a hospital needs to respond to a data breach, the more expensive it tends to be, and the longer the remediation process is, the more it costs as well [14]. It is not just the cost of a ransom payment, but also the loss of business during downtime, the post-breach response, the damage to the institution’s reputation that drives away the patient base, and the legal ramifications of compromising patient confidentiality [14].

Larger teaching hospitals are more likely to be subject to data breaches, which have been attributed to many reasons [10, 15]. There is more patient data to be gained by attackers, larger facilities with more equipment, more end users with access who may not have sufficient training or motivation to be secure, and more frequent staff turnover, particularly with residents and fellows cycling through the institution [15]. That does not mean that smaller hospitals are more secure, as there is the consideration of smaller IT teams and tighter budgets for system upgrades and new equipment in smaller hospitals.

Patient impact is also important. The effects of identity theft and credit card fraud committed with stolen data can be weathered by some individuals, but not all. Low-income or fixed-income homes are often disproportionately affected by data breaches. Similarly, vulnerable communities like children, people of color, or the elderly are also less able to recover from these costs [34]. For communities of color that already have a deep-rooted distrust in the healthcare system, fallout from a data breach can reinforce existing biases and reduce the use of lifesaving services. When children are affected, it can impact their future education by reducing eligibility for student loans or pushing them into predatory loans where there may be even more long-lasting financial consequences [34]. There are also legal or social consequences of health records leaking that could disproportionately affect women and marginalized individuals in certain regions of the United States.

Education and training of healthcare employees, including perfusionists, is necessary to improve cybersecurity across the board, but there is almost no consensus on how to do this effectively [8]. Employee engagement in training improves with material tailored to their proficiency levels and technical needs [5, 10]. Perfusionists should know common sources of cybersecurity risk in their field, such as legacy medical devices and phishing emails. Shared ownership of responsibility for cybersecurity helps maintain resilience in this constantly evolving field [5, 10].

Without employee buy-in, people will find workarounds for whatever is most cumbersome, bypassing security because they do not understand its necessity, and because ease of access is important in many healthcare situations [3, 6, 9]. To protect the integrity of both the hospital network and patient data, perfusionists need to know how to identify suspicious emails and have a clear understanding of their hospital’s data handling policy [4]. Perfusionists need to be empowered to view their space with an eye for digital security. In case their hospital was to become the target of a cyberattack, perfusionists should also be able to revert to paper documenting and be able to provide patient support without connected technology. They should also know that third-party vendors can be affected by cybercrimes and how that could impact a perfusion team’s day-to-day operations. Perfusionists in charge of purchasing should know what to look for in cyber-secure devices and be aware of what protections are legally required in devices. They should also know that there are ways to get involved with governmental cybersecurity policy and what training material is available for those interested.

It is easy in this day and age to look at the number of records reportedly already on the dark web or in the hands of foreign nations and jump straight to a pessimistic approach to data security, that it is already too little too late. But it is imperative to remember that this is a hidden, critical aspect of patient safety. Every new data breach is a new point of weakness in the digital health of our patients. While some individuals will have the time and resources to weather the consequences of identity theft or insurance fraud, there will always be those without those resources, for whom the fallout of a hospital data breach can be catastrophic, and it is the job of all healthcare workers to do their best to protect their patients’ data.

## Getting to know the players

### Government

 Government legislation is a powerful motivator for cybersecurity improvements, as avoiding regulatory, financial, and legal trouble through compliance can help motivate institutions to keep up with current cybersecurity trends [1]. While it is the government’s role to enforce legislation, it is also responsible for investigating, stopping, and prosecuting cybercriminals, across borders if necessary [10], as well as for setting criteria and defining boundaries within which the law applies. This is particularly critical with the complexity of medical devices and new artificial intelligence (AI) software applications in the medical field. Table 2 lists several relevant definitions for medical devices. With the increasing dependence of all industries on technology and the associated risks of cyberattacks or cyberterrorism, proactive public policies are a necessity [17].

**Table 2:** Technical definitions.

A medical device is defined in the Federal Food, Drug, and Cosmetic Act (FFDCA) of 1938 as an: [3]	Instrument, apparatus, implement, machine, contrivance, implant, in vitro reagent, or similarly related article. Includes any component, part, or accessory intended for use in the diagnosis of disease or other condition, or in the cure, mitigation, or prevention of disease. In man or animals.
According to the FDA, a device is considered a cyber-device if it meets all three of the following: [35]	Includes software validated, installed, or authorized by the [manufacturer] as a device or in a device. Has the ability to connect to the internet. Has any such technological characteristics validated, installed, or authorized by the [manufacturer] that could be vulnerable to cybersecurity threats.
“Connected to the Internet” if it can be defined as having any of the following, regardless of whether the connection is disabled: [36]	Wi-fi or Cellular. Network, server, or cloud service provider connections. Bluetooth or Bluetooth low energy. Radiofrequency communications. Inductive communications. Hardware connectors capable of connecting to the Internet (USB, Ethernet, serial port, etc.).
Three types of software related to medical devices are: [37] Any of these types that are “necessary for safe and effective use of a device” are termed “critical software.” [38]	Software which is its own medical device and is defined as “software intended to be used for one or more medical purposes that perform these purposes without being part of a hardware medical device”. Software related to and integral to a medical device Software used in the manufacture or maintenance of a medical device.

### Third-party vendors

Third-party vendors such as business associates, supply chain vendors, and medical device manufacturers (MDMs) are a major source of cybersecurity risk for healthcare institutions. Hospitals use many suppliers and often exchange information with them over years of working together [2]. Third-party vendors may or may not have access to protected health information (PHI) or personally identifiable information (PII), but because of their relationship with healthcare employees, any data breach on their end could result in malicious code being sent along legitimate channels to affect a hospital. They also may not have stringent cybersecurity, as the legislation surrounding their business practice does not necessarily fall within the same regulatory pool as healthcare facilities, making them a potential liability to their partner healthcare systems.

According to the American Hospital Association (AHA), third-party breaches can be some of the most disruptive, because a vendor can be a common link between many hospitals or hospital systems, and can affect each of those partners simultaneously [39]. This type of strategy, when performed specifically to access a secondary target or targets, is called a “hub and spoke” strategy, and it is increasing in popularity amongst criminals [39]. Hospitals can place defenses from third-party breaches by ensuring that they have incident response plans, alternative supply sources, and good business associate agreements that require cybersecurity and cyber-insurance for all parties, including any subcontractors the vendor chooses to employ [39].

Third parties who supply medical devices have a second source of risk in the form of the device software inherent to their product. MDMs often generate their own proprietary internal software and can be reluctant to share access to it, even with individuals intent on making the product more secure, such as hospital IT teams [6, 7]. However, as such, it is imperative that MDMs provide their own cybersecurity on the devices in question [6]. Up until March 29, 2023, it was not a legal requirement that they do so. And even past that date, only new products going through the Food and Drug Administration (FDA) approval process are required to have reasonable cybersecurity measures, and to provide upgrades and patches to this security throughout the lifetime of the device. More worryingly, MDMs are not required to provide upgrades and patches to pre-existing or legacy devices, though some may choose to do so.

### Information technology teams

Healthcare IT teams, though broadly understaffed and underfunded, are the primary implementers of cybersecurity measures around healthcare infrastructure. As with many other aspects of the healthcare field, IT teams have developed over time, and with the needs of new technologies. Current advancements that make healthcare IT teams more effective include the use of AI to monitor and analyze network use. This is a reasonably useful and cost-efficient method of increasing security for the time being [2, 14, 31].

### End users

The average user of healthcare-related devices or systems is often the weakest link in the cybersecurity system, especially if unprepared or undereducated [9]. This group consists of everyone from administrators and their assistants to perfusionists, surgeons, nurses, environmental service employees, and patients themselves. Though each of these groups will have access to different parts of the overarching hospital network, they all may serve as gateways through which cybercriminals may attempt to gain access to the network. Network segmentation, implemented by the hospital’s IT team, can help keep individual end users from becoming too large a risk, but education and vigilance are the best tools that employees can utilize to help IT personnel keep everyone safe [2].

### Insurance companies

The emerging field of cyber insurance is in response to the increased threat and costs of a cybersecurity breach. Much like any other insurance company, they help cover costs should a covered event occur, but in this case, they can help pay for things like fines, penalties, litigation defense, the costs associated with notifying affected patients, and supplying them with identity and credit monitoring [9]. While this does help mitigate the financial consequences of cyberattacks, it does not change the loss of personal data and privacy that the patients experience [6].

### Media

The media can also play a role in cybersecurity advancement. Media attention can be a double-edged sword, as it can raise awareness and pressure institutions or governing bodies to take action to make healthcare more cybersecure, but it can also spread distrust for specific institutions or in the healthcare system entirely [4]. While hospitals may assume that negative media attention will erode their patient base in the short term, it may be worse in the long run to be caught hiding a cyber-breach [4].

### Cybercriminals

There are many reasons a group or individual may resort to cybercrime. A common way of sorting this group is by motivation, the primary of which tends to be financial gain. There are several means by which financial gain can be achieved from a hospital cyberattack. If a hospital is held for ransom, they either gain ransom money or, if the ransom is not paid, cybercriminals may choose to either sell the hospital data, including patient information, on the dark web. These transactions are often completed in cryptocurrency, which makes them difficult to trace [40]. Data that ends up online is sold at a premium price.

Health information is more valuable than other types of identifying information [3, 5, 6, 10, 11], primarily because it is immutable. Financial information can be changed, some identifiers can be locked down, but genetics and physical health data cannot be altered by the individual in question. A full medical record often comes with a variety of other data as well, from financial information to names, social security numbers, phone numbers, email and physical addresses, and insurance numbers [7]. This data can be used to commit identity theft or fraudulently acquire health insurance benefits, such as prescriptions, which can be resold online [2, 6]. It can also be used to blackmail high-profile individuals [2, 7]. Often, money gained by a developed ransomware group essentially funds future cyberattacks on other institutions. There is always the possibility that the ransomware group will not release the decryption key regardless of payment, as happened to Change Healthcare in February of 2024 [24]. There are several well-known Russian ransomware gangs, and while they appear to be the largest single nation source of cybercrime, China, Ukraine, and the United States are reportedly frequent sources as well [41] (Tab. 3).

**Table 3:** Targeted data and goals.

PII and PHI targets	Name, date of birth, address, phone, social security number Bank account information Insurance information Health history	Sale on the dark web Identity theft Insurance fraud Blackmail
Non-PHI targets	Intellectual property (IP) Research	IP theft

A second motivation for cybercrime is espionage. Nation-states can commit or fund acts of cybercrime against healthcare institutions to gain insight into opponents, sow distrust in the government and public systems, and fund and fuel their own political agendas [5]. Occasionally, state-backed cyberattacks have been known to target healthcare facilities for their intellectual property, such as medical research [2, 42].

Hacktivism is a source of politically motivated cybercrime that is often not aligned with overarching governing bodies. They often have a particular political or theological motivation [27]. As such, hacktivism can be much smaller-scale than other politically motivated attacks, and can be internally generated within a country [6].

A mildly unacknowledged source of cybercrime is one with no malicious intent, but simply because an individual wanted to prove that they could. Prior to the availability of broadly available vulnerability disclosure processes through MDMs, individuals who tested the cybersecurity of devices or systems for this reason could and would be prosecuted [27]. This would result in cyber-savvy individuals knowing that there was a security flaw in a device but being unable to safely communicate that information, leaving the vulnerability for malicious hackers to find and abuse. Medical devices can now be tested, and flaws can be acted on in a collaboration between hackers and MDMs.

Cyberattacks could easily tamper with patient data, resulting in delays of life-saving treatment or incorrect diagnoses [2]. Though no cyberattack has yet been proven to be motivated by patient death, cyberattacks have been linked to patient deaths in hospitals through a broadly increased 30-day mortality rate, and also directly, as happened in Germany in 2020 when a woman was transferred out of Düsseldorf University Hospital while it was held by ransomware, and did not survive to surgery [43]. A second potential cybercrime mortality was a baby in Alabama in 2021 who was born while Springhill Medical Center was using paper charting due to an attack. In the confusion, the care team missed an abnormal fetal heart rate, allegedly resulting in the infant’s death [44, 45].

### Cybercriminal methodologies

Ransomware is a common type of cyberattack, but there are many other types of attacks. Classification of these is best left to the experts [46], but descriptions and examples of some of the more common types can be found in Table 1. Table 4 describes three of the most common access points for cyberattacks. These access points are of concern to all end users, including perfusionists. Phishing and other social engineering-based attacks are the most common, but with education and understanding of hospital policy, a healthcare worker can reduce the chance of precipitating a data breach in this manner.

**Table 4:** Common points of access for cyberattacks.

Category	Method	Example
Social Engineering (e.g., Phishing, Spear Phishing)	Use of personal information, sometimes gathered from social media, to convince an individual to give malware access to a system, or to give an interloper credentials for that purpose. Spear phishing is a more individually targeted version of Phishing.	Kaleida Health (2017) [9]
		Premera Blue Cross (2014) [18]
		Anthem, Inc. (2015) [18]
		University of Vermont Health Network (2020) [4]
Malicious Insider	Deliberate action by an individual with valid institutional access to access data, encrypt systems, or inject malware into a system, or to release sensitive data they have access to onto the internet.	
Carelessness	Accidental failure of an individual who has valid access to a system to adhere to the security policies, or lax policies allowing excessive access, amongst others, means of unintentionally reaching data the individual should not access.	Cerebral third-party data breach (2023) [18]

Most successful cyberattacks use known exploitable vulnerabilities (KEVs) and work because of a lack of updated security policies that would have prevented such an attack [2]. Public Wi-Fi is another point of concern, as they often have readily available or easily guessed passwords. Even with secure Wi-Fi, a common tactic to gain access to a hospital network is to simply ask for log-in credentials off a link in a phishing email [2]. This gives an attacker a gateway to more secure systems that often have unsecured IoMT devices attached to them.

There are, of course, many other types of ways that hospitals can come under attack that have nothing to do with any motivation directly against the hospital itself. Attacks that are so broad-spectrum that they simply catch healthcare systems in the crossfire, such as the WannaCry attack [17]. The recent CrowdStrike downtime had an impact similarly wide-reaching, even though it was not a deliberate malicious attack. Being prepared to face cyberattack-based downtime should prepare a hospital to deal with this kind of less malicious interference as well.

## The current state of healthcare cybersecurity in US governmental policy

Legislation for cybersecurity over the last decade and a half has been primarily reactive. Due to the lack of core planning at the outset, there has been an ad-hoc method of establishing jurisdiction and oversight. Three major US Departments have jurisdiction over cybersecurity that is of interest to perfusionists. These include the Department of Homeland Security, the Department of Health and Human Services (DHHS), and the Department of Commerce. Each of these departments oversees a different organization or agency that has established ways in which cybersecurity is regulated related to their domain of expertise. Within the last few years, there has been a significant increase in governmental interest in establishing solid foundations for all national cybersecurity needs, but it is still very much under development. Table 5 covers a brief synopsis of each department as well as the influence of both the Health Insurance Portability and Accountability Act (HIPAA) and the Health Information Technology for Economic and Clinical Health (HITECH) Act that came before them.

**Table 5:** Overview of healthcare cybersecurity in US governmental policy.

Policy or policymaker	Effects on cybersecurity that may relate to perfusion or healthcare.
Health Insurance Portability and Accountability Act (HIPAA) (1996)	Arguably the first step in healthcare cybersecurity. Requires that relevant entities maintain reasonable and appropriate safeguards for patient privacy in three different fields: administrative, physical, and technical [6, 47]. Cybersecurity is not explicitly named, but does have a strong basis in this federal law, and many future policies and guidelines were built off of this foundation.
Health Information Technology for Economic and Clinical Health (HITECH) (2009)	Built upon the privacy and security provisions of HIPAA, but it primarily outlines penalties and enforcement of HIPAA rules by the US DHHS Office of Civil Rights [48]. Data breaches >500 individuals must be reported within 60 days of discovery [9, 15]. Fines ranged from $137 to $68,928 per violation of HIPAA. Maximum $2,067,813 per hospital per year in 2023 [49]. Does not add to HIPAA’s technical safeguards or encourage advancement in cybersecurity. Set financial incentives for healthcare providers to establish “meaningful use” of an electronic medical record [6], and kick-started the mass conversion to digital record keeping. EMRs do improve patient care [10]; however, the mass implementation of them also made patient data vulnerable through encouraging enormous patient databases with little to no cybersecurity guidance [9].
National Institute of Standards and Technology (NIST)	Falls under the umbrella of the US Department of Commerce Hosts the National Vulnerabilities Database, a record of all governmentally known cyber vulnerabilities. This database can be accessed by both MDMs and IT departments to receive updated information on the latest threats they may need to patch [9]. Released the first Framework for Improving Critical Infrastructure Cybersecurity in 2014, based on Executive Order 13636 [50]. The framework outlined ways that industries could manage cybersecurity risk, without placing any regulatory requirements on those industries [51]. The framework was updated in 2017 and 2018 with better clarification for industries, though the implementation of the guidelines remained voluntary.
Food and Drug Administration (FDA)	A subsection of the Department of Health & Human Services. Generates pre- and post-market guidance for MDMs regarding cybersecurity. Updates these guidance documents every few years. New pre-market guidance slated for 2025 to better define which devices are required to comply with cybersecurity regulations [36]. Similar to the NIST framework, pre- and post-market guidance is nonbinding and unenforceable, but could delay a product going to market if not taken into consideration. The Food and Drug Omnibus Reform Act (FDORA) of 2022 mandated cybersecurity for any device that meets the definition of a “cyber device” (Table 3). Went into effect for any medical device seeking approval for market after March 29, 2023. FDORA requires that MDMs plan to support cybersecurity updates throughout the lifecycle of the product, whenever risks, threats, and vulnerabilities are discovered that may affect the device. Patches must be available on a “reasonably justified regular cycle”. FDORA also requires that all MDMs have a method of coordinated vulnerability disclosure, which often amounts to a location on their website for individuals to report cybersecurity vulnerabilities to the company, so that they may be addressed [36].
Cybersecurity & Infrastructure Security Agency (CISA)	A part of the US Department of Homeland Security that oversees sixteen sectors that have been deemed critical infrastructure, including the Healthcare and Public Health sector. Currently endeavoring to place the burden of cybersecurity on the tech providers and software developers rather than the consumers [52], but with an extended scope to help secure software supply lines and thus reduce risk for the economy and national security [52]. Works to smooth out information sharing between federal and private sectors, in line with a 2021 Executive Order on Improving the Nation’s Cybersecurity (EO14028) [53]. Defers to the US DHHS as its risk management agency. The DHHS has not updated the sector-specific plan since 2015 and defers back to NIST’s Framework.

For perfusionists, the DHHS also coordinates the Health and Human Services Cyber Performance Goals, which are a set of two levels of goals: essential and enhanced. These goals include the presence of an Incident Response plan as an “enhanced goal.” Vulnerability remediation planning, supply chain incident reporting, and vulnerability disclosure are also potentially relevant goals [54]. As of October 2024, there are also two proposed bills awaiting decision by Congress regarding enhancing the regulation of healthcare cybersecurity [55, 56].

Table 5 is by no means a comprehensive review of all vested parties with regard to cybersecurity and the healthcare field. There are many other organizational branches within the US government, as well as US nongovernmental organizations and international organizations that have regulations and guidelines. The FDA recognized the guidance from the Association for the Advancement of Medical Instrumentation in 2023 [57]. The International Standards Organization and the International Electrotechnical Commission are both referenced in many instances [8].

**Figure 1:**
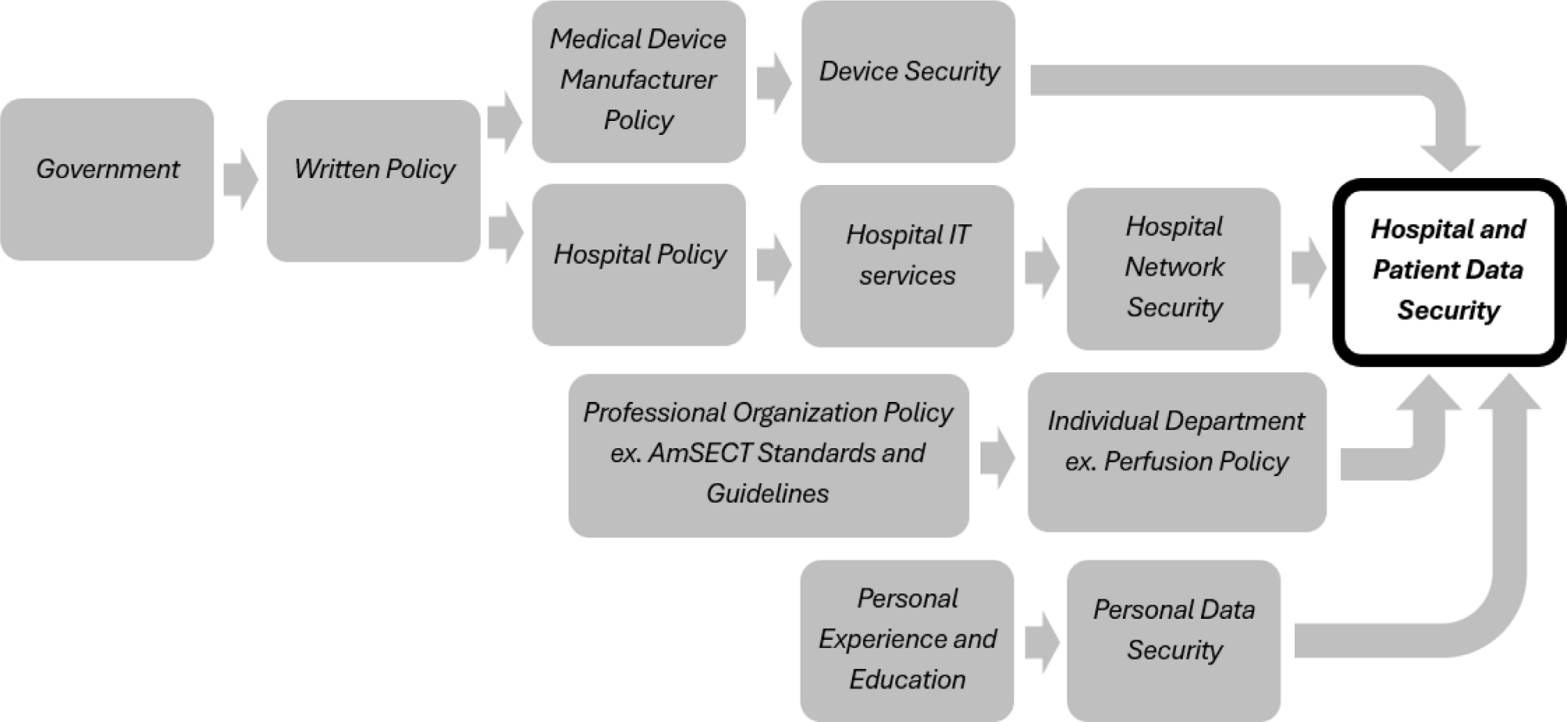
Simplified flow of cybersecurity policy to enhance hospital and patient safety.

In a 2024 article, Pourmadadkar, Lezzi, and Ardebili identified that cybersecurity risks are the primary threat to critical healthcare infrastructure [58]. Their study focused on risk associated with disruptions during a coronary artery bypass graft procedure (CABG) and specifically cited that a lack of legislation and policy on cybersecurity contributes to the severity of the risk that cyberattacks pose to CABG surgery [58]. While US legislation may be patchy, individual organizations have increasingly taken it upon themselves to empower individual professions. One example of this is the AHA, which has advice and programs that healthcare organizations can use to bolster their cyber defenses [39]. The interaction between sources of cybersecurity policy and security can be pictured in Figure 1.

## Current cybersecurity for perfusionists

Perfusionists already participate in many hospital-wide cybersecurity initiatives. Hospitals implement test phishing emails and yearly training on digital security and physical security with regularity. These are the basics that keep hospitals minimally safe. While it is imperative that competence, if not excellence, be displayed on these fronts, there are additional threats that may be attributed to perfusion or perfusionists from equipment and vendors, even without active knowledge of the hazards they pose.

### Perfecting the basics

- *Phishing* remains a very large threat to hospital systems, for a wide variety of reasons. Stress, burnout, and excessive emails all contribute to lowered ability to accurately detect phishing threats, whereas time, repetition, and personal experience all lead to higher detection rates among professionals [8, 10, 33]. It has become increasingly common practice over the last decade to have healthcare IT groups send test phishing emails to employees. These emails serve multiple purposes: first, alerting the employee in question as to whether they are being attentive and cautious in their online practices, and second, slowly introducing repetition and practice that employees need to maintain reasonable scrutiny long term. See Table 6 for common indicators of a phishing email.
- Hospitals also frequently have yearly online *training programs* focused on proper handling and disposal of protected information. They also cover physical security, such as not allowing strangers to tailgate through badge-access-only areas, since a cybercriminal could use this method to get to individual devices or secure network jacks. They also tend to reinforce the policy of having secure passwords and never leaving a computer unlocked, which are core components of digital security.
- *Cyber hygiene* is a set of practices that helps keep networks and data secure by reducing risk factors. This includes practices like good password management, a healthy suspicion of phishing emails, consistently updating software, and using antivirus software. The promotion of good cyber hygiene is a key way to promote digital security culture in the healthcare setting [7]. Beyond training with hospital practice phishing emails, and the general promotion of cybersecurity awareness, cyber hygiene includes an emphasis on reducing the leakage of information on social media platforms [8]. Information gathered online and used to target specific individuals for information is known as Spear Phishing, and can be even harder to detect than traditional phishing. Spear phishing emails can look more authentic and generally contain enough personal details to convince an unaware individual that they have a legitimate business or individual behind them. Some of the ways to combat this include limiting online presence, not posting any personal information, and going through the security settings of all social media apps and setting them to the highest privacy levels [8].

**Table 6:** Possible indicators of a phishing email [59].

Suspicious senders	The sender of an email should be legitimate, have the correct spelling and domain name for the topic, and should not be a spoof email. A spoof email sender will have a different email address when the cursor is hovered over the sender’s name.
Unnecessary or unwarranted urgency	Emails that seem particularly urgent play on stress to avoid scrutiny. Urgent emails out of the blue should be viewed with suspicion, particularly if they ask for account access to anything.
Too good to be true messages	If a message seems too good to be true, there is a high likelihood that it is. Caution when clicking on any links or attachments in these emails is warranted. Verification of legitimacy from an outside source is recommended.
Embedded or spoofed hyperlinks	The website associated with a hyperlink can be verified by hovering the cursor over the link. Ensure that the website you expect is the actual hyperlinked website before clicking on the link. Unexpected or gibberish links should not be trusted.
Grammar or spelling mistakes	Emails with spelling, grammar, or layout mistakes are likely not from large corporations’ marketing departments, but instead are knockoffs with malicious intentions.

### Raising awareness

The relatively sheltered and anonymous position that perfusion holds may have inadvertently allowed a lack of investment in digital education over the past decade. Hospital departments that were more vulnerable to cyber threats due to high levels of connectivity or media attention from high-profile security flaws have been under heavier scrutiny and have been the focus of security improvement because of that. Radiology, cardiology, anesthesia, endocrinology, neurology, and mental health all fall under this umbrella and have been the subject of research, education, and IT improvements [7, 11]. As other hospital departments, including surgery and perfusion, become more and more integrated, it is imperative that the lessons learned by others in the past decade be well utilized.

A major advantage that the perfusion profession has is the legislation that has been pushed through, and the prominent device cybersecurity failures that have motivated improvements in the device software. However, given the previously voluntary nature of medical device cybersecurity, there is a wide variety of compliance in devices. Current regulatory requirements place pressure on MDMs to add cybersecurity to new devices that were not previously covered. This is a burden that may cut into the profits of MDMs, and as such, it may be of interest to healthcare professionals and the general public to be attentive and hold them to the high standards that patients expect of their medical care.

Perfusionists and MDM product representatives should both be aware that monitoring of cybersecurity threats relevant to products FDA-approved after March 29, 2023, is legally required. Cybersecurity updates are legally required over the lifetime of the device, both on a schedule and when major vulnerabilities are found. A Software Bill of Materials (SBoM) is to be provided by the manufacturer to the hospital upon purchase of a product. This SBoM is a list of all software components, particularly those from subcontractors or outside parties, such as the Windows operating system. This allows the hospital IT department to track if updates or patches are necessary in the future.

### Discussing options

It is also in the best interest of perfusionists and healthcare centers everywhere to ask MDMs for cybersecurity patches or updates for legacy devices and older models of devices that do not have any cybersecurity but are still within the expected lifetime of the product. MDMs may be able to support the request, but even if they are not, it provides increased awareness among MDMs that these features are wanted and expected in products and could move the needle on what they focus on for the future. Perfusionists often are able to choose which products they need and which company they purchase from. As the primary customer interface for some very niche and expensive products, perfusionists have the power to drive the market in this way. The IT department of a hospital may be brought into a conversation about future product cybersecurity if a line of communication is established between the two departments.

For perfusion teams that use external perfusion record companies, there should be a similar inquiry regarding the digital security of the company that supports them. Like many other third-party healthcare vendors, if a cyberattack were to hit one of these companies, it could potentially wipe out the records of multiple healthcare centers, having an outsized effect on patient care. This could be particularly critical if the company also has the ability to remotely access equipment for software upgrades. If a remotely accessible pump or ECMO machine were interfered with during a third-party breach, there could be a direct and immediate effect on a patient’s life.

Beyond encouraging MDMs to consider supporting the software patching of older or legacy devices, another thing that perfusionists can ask for is the disabling of network ports on devices upon their retirement from support. Items that do not need to connect to the hospital network should not maintain that capacity if they are vulnerable. If this is not reasonable for patient care reasons, then working with IT to ensure that the device is properly isolated on the network is imperative.

## Next steps for perfusionists

What can individuals actually do to help with cybersecurity? There are plenty of recommendations available for IT experts, healthcare administrators, or researchers. Many of those recommendations even include the suggestion that every healthcare staff member be educated on cybersecurity, but there are few articles written for end-users of medical devices [3, 4], and end users are the ones who are most likely to be caught unawares with an IoMT device that is misbehaving, a phishing email or the clinical consequences of not being prepared for a system wide lockout. Healthcare employees are certainly not being asked to become experts in cybersecurity, but recognizing where there are modifiable risks regarding cybersecurity is of value to everyone [13]. Essentially, what can we do to better ourselves?

### Education

The first and most important step is to coordinate with all relevant departments. As perfusionists, we work with anesthesia, nursing, and surgery, all of whom are at varying levels of vulnerability, but could suffer the same consequences of being unprepared if anything were to happen to the hospital network as a whole. Ask questions and share information concerning the risks of intra- and interdepartmental devices. Coordinating questions and answers from the hospital IT team and supporting each other in learning is a great way to raise the bar across all departments. As much as 95% of data breaches within the healthcare field are from human error [2, 10, 13]. Every department within the hospital can stand to brush up on its knowledge of common threats and ways to prevent vulnerability.

It is also critical to know what personnel resources are available to the team and how to contact them [3]. If someone from perfusion notices a suspicious email, do they know how to report it to their IT team? If an anesthesia monitor does not seem to be properly reporting infusion rates or drugs to the patient’s chart, who gets called and by whom? If a legacy device is about to be connected to an EMR, and a team member has concerns about the risks of that, is there someone in charge of that who can be contacted? Are there MDM cybersecurity experts available to answer questions regarding medical devices? Outside of the hospital, who is the state’s local Cybersecurity & Infrastructure Security Agency (CISA) representative? CISA representatives are available for public outreach and education when requested, and there are educational resources and training courses available on their website. Should someone have serious concerns with the state of healthcare cybersecurity as a whole, are they aware that many FDA proposed guidance documents have a period of public comment online?

It is not taught, but equally important to know where individual system failure points could be, and what to do if those systems go down. If an external perfusion record company is taken out by a cyberattack that does not impact the hospital network, is the department prepared to go without it? Does the hospital have the ability to roll back an update on a pump if a new vulnerability is noticed, or to shut down remote access if there is a risk of malware unintentionally brought in from that third-party vendor? If the hospital is hit with a DDoS attack and devices fail to connect and emails do not go through, how will the relevant departments coordinate?

Recommendations that may be implemented by perfusionists or perfusion departments include:

- Practice simulations of cyberattacks or severe downtime incidents [3, 11].
- Store policies offline, particularly a cyber-incident response plan, should be stored offline and in hardcopy [3].
- Regularly back up department data and relevant software and store it offline [2, 3].
- Do not connect to public Wi-Fi [11].
- Do not leave devices that can access PHI unattended [11].
- Use multifactor authentication whenever possible [59].
- Educate all employees so that workarounds are not used to avoid cybersecurity [2].
- Do not access the web from critical devices such as downtime computers [3].
- Educate and re-educate employees to recognize phishing attempts and ensure they understand the potential severity of the consequences of not recognizing one [3].
- Reduce the risk of spear phishing attempts by utilizing the security settings on social media platforms and avoiding posting personal or job-related information [8].
- Put access controls on individual devices and ensure their time out [11].
- Revoke email distribution list access to individuals who no longer belong on it.
- Revoke client or user access from individual devices when it is no longer warranted [3, 59].
- Know what a reportable cyber incident is and who to report to [3].
- Open communication channels between IT personnel and individual departments to ease feedback and allow IT to address workflow issues if necessary [5, 11].
- Provide additional security training to individuals with access to privileged accounts [3].
- When involved in purchasing new devices, ask third-party vendors about their cybersecurity policies, how much cybersecurity support there is for their device, how long it lasts, and if they support legacy devices. Also inquire as to the security of their subcontractors or suppliers.
- When involved in purchasing, a Manufacturer Disclosure Statement for Medical Device Security (MDS^2^) should be required and provided to IT [4, 59].
- When involved in purchasing, ensure that a Software Bill of Materials is included and provided to IT [59].

Password-related advice includes:

- Do not reuse passwords.
- Do not share passwords.
- Change passwords frequently [11, 59].
- Do not use passwords that are one number or letter off from previous passwords [7].
- Use a 15-character minimum for passwords [59].
- Change any default passwords on devices before connecting the device to a network [59].

There is a running motto within cybersecurity fields that goes: “if you can’t secure the device, secure the network, if you can’t secure the network, secure the data.” Currently, much of IoMT device security is provided by MDMs, and unless the device went through the FDA approval process after March 29, 2023, there is no guarantee of any cybersecurity on it, so we must rely on hospital cybersecurity to protect the network and data instead. As older and legacy devices are replaced, however, this will change. For individuals who have the ability to choose which product to purchase, emphasizing the need for cyber-secure devices to MDMs will help drive the field forward. CISA has a series of recommendations for what to look for in devices. They highly recommend that manufacturers build with a “Secure by Design” standard, meaning that cybersecurity is built into all aspects of the software, not patched on after the fact. This will be visible in a few ways [59]:

- No default passwords
- Single sign-on option available
- Security audit logs available
- Defaults to high security settings

Most importantly, none of these should be add-ons that cost extra. They should be a part of the default package [59]. An add-on indicates that cybersecurity was not a part of the software and product design from the onset, and instead is a patch or afterthought, which makes it more vulnerable.

Finally, for education, a direct supervisor has more of an impact on employee buy-in and compliance than any top-down leadership directive [12]. Supervisors or team leaders can encourage individuals to take an interest in cybersecurity, develop a team member into an IT coordinator role, and educate them when capital purchases are available to interrogate. To make actionable changes in a workforce and mitigate 70% of inadvertent data breaches that are caused by employee accidents, there needs to be a cultural shift, employee engagement, and encouragement by individual department leaders [12]. The recommendations made here can certainly be implemented by individuals, but managers have the additional option of endorsing policy changes that have a broader impact and are less likely to be ignored than warnings or actions of a single person. Similarly, endorsement of increased cybersecurity by governing bodies such as AmSECT would increase the reach and significance of the message and embolden professional growth. An example of a possible Standard and Guideline might be:

- Standard: The organization shall ensure cybersecurity for all new perfusion hardware devices to the best of its ability, and review cybersecurity risks annually.
- Guideline: Clinical personnel should have a procedure to operate in network downtime and be able to coordinate with relevant departments to ensure patient safety in the absence of an electronic record.

### Asset lists and risk management

One step that a perfusion department can take to immediately improve its understanding of cybersecurity risk is to generate an asset list. This is a summary of devices, their software version, update history, associated components, and method of network connection. This is useful for vulnerability management and patch management [3]. For perfusionists, the hospital IT team should already have completed an asset list that includes their devices, but it does not hurt to double-check. There can be instances where devices fall through the cracks, particularly smaller device purchases that may not meet capital equipment status. The IT adage “you can’t secure what you can’t see” rings true here.

A second common IT assessment that is done behind the scenes is a Risk Assessment. A risk assessment is one of the first things an IT team does when assessing cybersecurity. This involves establishing how each device is connected to others, how much of a threat each one poses to the overarching system, and if there is anything that can be done to mitigate or isolate those risks [2, 9]. A discussion between perfusionists and their hospital’s IT team to ensure that the severity level of each device’s risk is understood could be beneficial if the IT team has time and resources to do so.

These IT techniques are not solely applicable to cybersecurity. Creating a departmental asset list and having knowledge of unprotected legacy devices readily available may also help to drive device turnover. It could also be used to isolate which device manufacturers need to be contacted regarding any available security patches. Keeping up with updates can be one of the biggest challenges of medical devices, and oftentimes, a hospital can employ an individual who tracks updates posted to MDMs’ websites [37], but especially with niche devices as are often found in perfusion, this may be a tall order.

A department-specific risk assessment would also be of use. Identifying which device functions are most critical, what alternatives are available, and which need to be prioritized for isolation and repair removes some stress during an actual crisis. Together, these two techniques essentially form preemptive technological triage, limiting delays during an incident.

### Emergency preparedness

Whether it be organized specifically as a business continuity plan in case of a cyberattack, or simply covered under “downtime policies,” perfusionists should have a plan for operating without critical information systems or connectivity between systems. These plans should be available as a hard copy or at least be available from an offline source. There should be some agreed-upon method for paper charting, not only for perfusion but for nursing and anesthesia as well. Drills or shadow charting should be run at regular intervals to ensure that all team members are familiar with this backup option. This practice helps provide a smoother and more complete transition to backup measures during an incident, minimizing the impact it has on essential services [4].

Backups of data and software should be made at regular intervals and stored offline [2]. Perfusion-specific policies or an individual’s work-based projects can be reasonably stored long-term by the department, whereas patient data should be backed up by the hospital or EMR service. This practice is helpful not only in the event of a cybersecurity incident, but also in case of unintentional loss of data from events like corrupted drives.

### Additional considerations

A special consideration that may not apply to all perfusionists, but warrants discussion, is the vulnerability that carries over to international communities who rely on outsourced technologies or services. Cybersecurity is not simply a need of developed countries or world powers. Cybercrime can occur anywhere with technology or data, and all of the same sources of vulnerability may apply in places that perfusionists may travel for mission trips, international outreach programs, or disaster relief efforts. Equipment concerns, particularly for older equipment donated for overseas use, arise because these items are often well past their warranty, and beyond service updates, not only for the physical components but also those of the onboard software. Networking these devices may not be as common in some areas of the world, thus limiting the breadth of the local IoMT, but that does not mean that databases are safe, nor does it indicate that those devices will never be added to a centralized system later.

Outreach groups are also similar to other third-party hospital connections. They can be a source through which vulnerabilities may be brought into a hospital if cybersecurity is compromised on the side of the partner. This could work similarly to other hub-and-spoke attack strategies, simply with fewer targets. This should not be considered a deterrent for assisting other centers, but a reminder regarding vigilance and the importance of sharing cybersecurity concerns and best practices.

Finally, all of the policies reviewed here are from the perspective of the United States, though many of the cybersecurity concerns and preventive measures discussed are applicable on a more global level. For additional information from other governments, this author suggests reference [60] as a potential starting point.

## Conclusion

Cybersecurity is a difficult field to explain and educate in fields outside of IT, despite how crucial it has become in the modern era. Perfusionists should also know what kind of impact a cyberattack could have on them, their workflow, and their patients, so that they may best mitigate the impact or isolate the vulnerability. Planning for worst-case scenarios is a part of a perfusionist’s job, and that should include preparation for a lack of technology. Each perfusion department should have a plan for operating with limited or no device connectivity in case of a system outage, a cyberattack, or otherwise. The increasing interest in cybersecurity across all industry sectors indicates that it may be prudent to work towards an AmSECT standard or guideline, thereby encouraging discussion of digital safety and keeping the profession in line with other medical societies.

Forming a digitally secure and cyber-resilient perfusion department is not a simple objective. Technology will only become increasingly integrated into the personal and professional lives of perfusionists and patients. Healthcare as a whole has already seen the consequences of not keeping ahead of digital security, with cyberattacks now costing billions each year. The perfusion profession should strive to learn from healthcare system attacks of the past and be a part of the solution. Much like the cultures of safety, diversity, and inclusion that have been cultivated over years of active policy shifts within healthcare institutions until they become commonplace, so too must we promote a culture of digital security, enacted and encouraged by each and every employee, for the betterment of our workplace and our patients’ safety.

## Data Availability

No data was created or analyzed in this study. Data sharing is not applicable to this article.
